# A High-Strength, Absorbable, Antibacterial Knotless Tissue Control Device for Fascial Closure

**DOI:** 10.1007/s13669-017-0208-0

**Published:** 2017-05-06

**Authors:** Jesse G. Nawrocki, Heather Nonnenmann, Mark Mooney, Nadia Sutton, Niels-Derrek Schmitz

**Affiliations:** 1 Johnson & Johnson Medical Devices, Somerville, NJ USA; 2 Johnson & Johnson Medical Devices, Norderstedt, Germany

**Keywords:** Barbed sutures, Knotless tissue control device, Stratafix™

## Abstract

**Purpose of Review:**

This review provides an overview of the STRATAFIX™ SYMMETRIC PDS™ Plus Knotless Tissue Control Device design and performance characteristics and highlights the device’s relevance for use in gynecological procedures. Various device testing was conducted on tensile strength, fixation tab mass comparison to conventional suture knot tower, initiation stitch strength, and wound holding strength to highlight the STRATAFIX™ SYMMETRIC PDS™ Plus Device’s key product attributes that may benefit general and minimally invasive gynecological procedures.

**Recent Findings:**

This article serves as a technological assessment of the latest barbed suture offered by Ethicon—STRATAFIX™ SYMMETRIC PDS™ Plus Knotless Tissue Control Device. This device is indicated for soft tissue approximation and can be used to close high tension areas, such as fascia.

**Summary:**

Barbed sutures were successfully introduced to gynecologic surgery many years ago, and their safety and effectiveness have been demonstrated in a variety of gynecological surgical procedures. By eliminating the need to tie surgical knots, barbed suture provides a few key advantages over conventional suture, such as reducing operating room time, eliminating potential knot-related complications, and reducing suturing difficulty in open and minimally invasive gynecological procedures. Additionally, there are tensile strength and wound holding strength advantages (vs. conventional PDS™ Plus Suture) described in the product testing highlighted in this review that may be relevant for gynecological procedures.

## Introduction

There is a growing body of evidence regarding the safe and effective use of barbed sutures or Knotless Tissue Control Devices in general and minimally invasive gynecological procedures. Barbed sutures, which allow consistent tension control over the suture line and avoid the need for knots, were first used in gynecologic surgery by Greenberg and Einarsson in 2008 [1•]. These devices are effective for reducing procedural time, achieving superior hemostasis and comparable wound approximation to traditional sutures.

Ethicon has a portfolio of two technologies in the market: STRATAFIX™ Spiral and STRATAFIX™ SYMMETRIC Knotless Tissue Control Devices. These devices eliminate the need to tie surgical knots and can reduce operating room time and potential knot-related complications. Additionally, by eliminating knots, the STRATAFIX™ Knotless Tissue Control Devices reduce suturing difficulty, especially in minimally invasive gynecological procedures. The barbs on these devices aid in achieving an intimate closure and enhanced tissue engagement with each pass.

A variety of studies have proven that barbed devices such as STRATAFIX™ Spiral have similar or improved outcomes as compared to traditional sutures. The safety and effectiveness have been established in dermal closure of non-emergent Pfannenstiel incisions [2, 3], closure of the uterus in laparoscopic myomectomies [4, 5, 6, 7], vaginal cuff closure in total laparoscopic hysterectomies [8, 9], and in robotic sacrocolpopexy [10]. Isolated cases of bowel obstruction have been associated with the use of barbed sutures during laparoscopic surgery.

The purpose of this paper is to provide an overview of the design and performance characteristics of the new knotless tissue control device STRATAFIX™ SYMMETRIC PDS™ Plus. This device is the first barbed suture that can be used for the closure of high tension areas, like fascia. This device has a novel barb design (anchors) to facilitate high strength soft tissue approximation along with the performance and absorption characteristics of PDS™ Plus Suture. The added features and benefits of the anchors may increase the efficiency of tissue approximation while ensuring the appropriate wound support during healing. As with PDS™ Plus Antibacterial (polydioxanone) Suture, STRATAFIX™ SYMMETRIC PDS™ Plus Device is used in many surgical applications such as fascial closure where an absorbable suture offering extended wound support (up to 6 weeks) is required [11•, 12•, 13].

### Device Design and Characteristics

STRATAFIX™ SYMMETRIC PDS™ Plus Device is a novel, absorbable, antibacterial, Knotless Tissue Control Device developed to facilitate soft tissue approximation by providing the performance characteristics and wound holding security of conventional PDS™ Plus Suture with added features that offer increased efficiency and control. The device is composed of polydioxanone, which is identical in composition to the absorbable polymer material that is used to produce PDS™ Plus Suture.

**Fig. 1:**
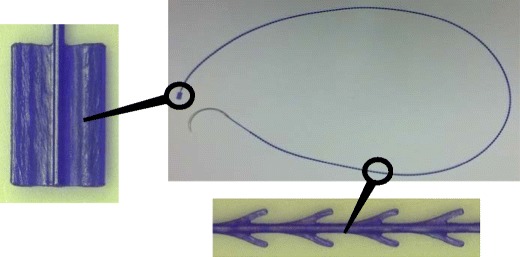
Example of a STRATAFIX™ SYMMETRIC PDS™ Plus Device

The STRATAFIX™ SYMMETRIC PDS™ Plus Device is illustrated in Fig. 1. It features a solid core with a series of unidirectional anchors evenly spaced down the length of the device in pairs symmetrically orientated 180° from each other. The strength and integrity of the core are maintained as the anchors are integrally formed onto the core.

The size and spacing of the anchors is uniquely designed to provide maximum holding in soft tissue where PDS™ Plus Suture is commonly used, such as fascia. The anchors are also designed to provide tactile feedback during passage through tissue, which helps the surgeon apply the desired tension. Each pass of the device maintains the tissue approximation without the need for an assistant to hold tension or “follow the suture” as is necessary when sewing with a continuous or running technique using a conventional suture.

The distal end of the device has a unique feature described as a fixation tab that anchors the first pass into tissue. The fixation tab eliminates the need for a surgical knot to anchor the proximal end of the incision. The device is implanted using a continuous suturing pattern and requires only minimal changes in the technique used to implant conventional sutures. The only significant technique differences are at the ends of the suture line, as described in the next section.

This new device is also treated with IRGACARE ® MP (triclosan) which inhibits bacterial colonization of the device as it does with traditional PDS™ Plus Suture [14]. STRATAFIX™ SYMMETRIC PDS™ Plus Devices have been proven in vitro to kill bacteria on the device that are commonly associated with surgical site infections and to create zones of inhibition [15].

### Description of Intra-operative Usage

**Fig. 2:**
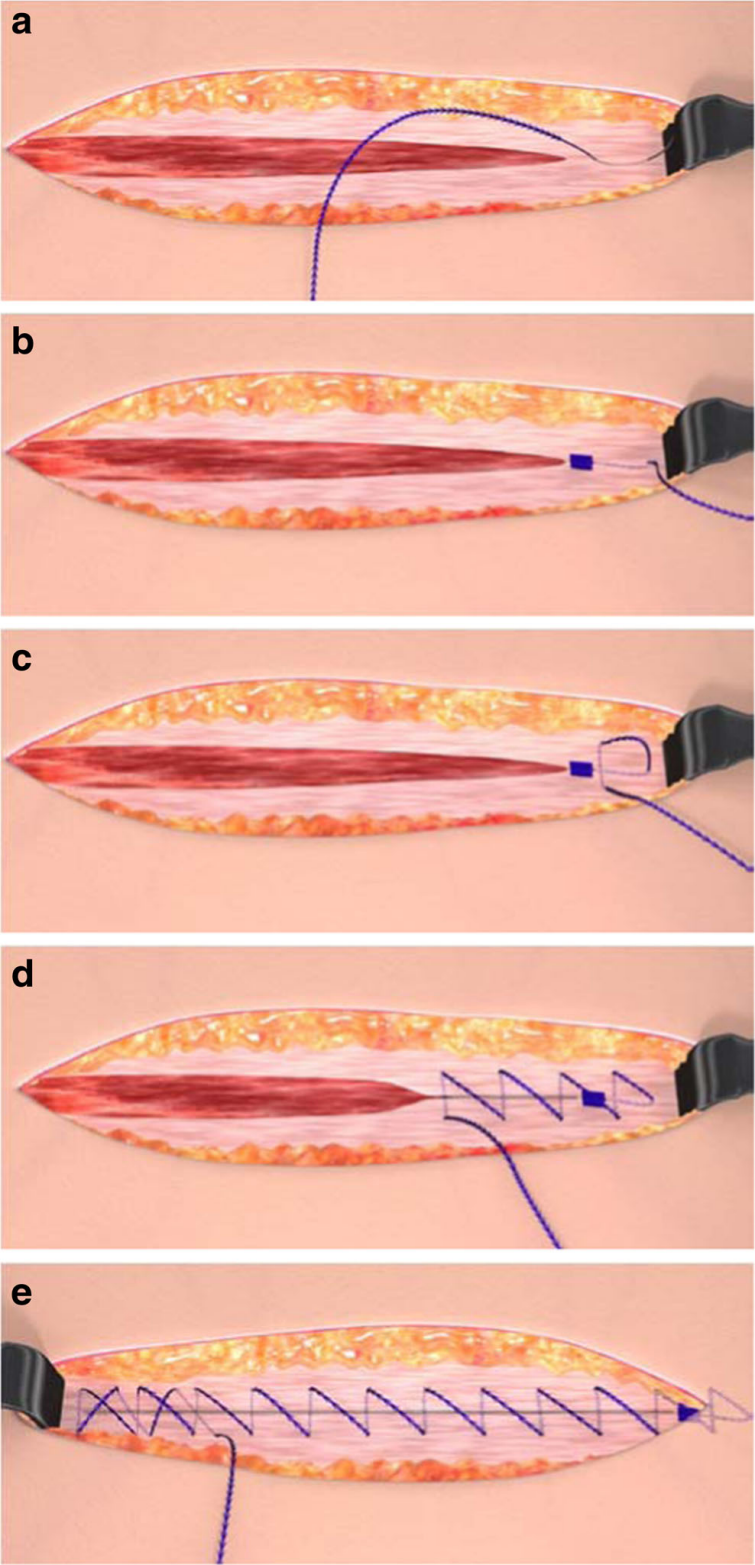
Illustrations of the basic steps to close an incision using STRATAFIX SYMMETRIC PDS™ Plus devices

STRATAFIX™ SYMMETRIC PDS™ Plus Devices are designed to be used in continuous suture patterns without anchoring knots at the beginning or end of the closure line. The basic steps are illustrated in Fig. 2a–e. To begin the closure, take the first pass directly above or adjacent to the apex in a direction away from the incision (2a). Pull the device through the tissue to gently seat the fixation tab. The fixation tab should be seated above the tissue plane and be visible (2b). Moving toward the apex of the incision, take a pass in the intact tissue perpendicular to the initial pass to lock-in the fixation tab. Multiple passes are acceptable (2c). Proceed with a continuous suturing pattern to close the incision, taking apposing bites on either side of the wound in standard fashion. To achieve the desired approximation and tension, gently pull on the device with each tissue passage (2d). Over tightening of any suture can lead to ischemia and necrosis. To complete and secure the closure, take two passes in the reverse direction across the incision. Finally, gently pull on the free end of the device and cut flush with the surface of the tissue (2e).

This technique provides a balanced closure for STRATAFIX™ SYMMETRIC PDS™ Plus Devices from end to end. Locking the fixation tab into tissue at the proximal end mimics the pair of reverse passes at the distal end. The unidirectional anchors lock into tissue with each pass along the length of the closure to achieve intimate and secure approximation.

### Device Performance and Evaluation

#### Tensile Strength

Suture strength is characterized by the maximum tensile load of a simple knot throw as per USP requirements [16]. It is well established that the knot is the weakest point in an implanted suture [17]. Since the STRATAFIX™ SYMMETRIC PDS™ Plus Devices require no knots to approximate tissue, the maximum tensile load of the core for the STRATAFIX™ SYMMETRIC PDS™ Plus Devices was compared to the maximum tensile load of equivalent sized PDS™ Plus Suture knots.

The tensile strength of the STRATAFIX™ SYMMETRIC PDS™ Plus Devices was evaluated using a standard Tensile Testing method. A 4-in.-long specimen was fixed at a set gauge length using steel-faced pneumatic clamps. Specimens were loaded at a constant strain rate until rupture using a calibrated INSTRON™ Mechanical Testing Unit.

The tensile strength of the PDS™ PLUS Suture was evaluated using the same method described for STRATAFIX™ SYMMETRIC PDS™ Plus Devices with the exception of a simple knot introduced into the center of the specimen length prior to loading into the INSTRON™. This testing methodology is established in the reference standard 881 for Suture Tensile Strength issued by the US Pharmacopeia [16].

Twenty samples per group were tested. The maximum tensile loads of the STRATAFIX™ SYMMETRIC PDS™ Plus Devices are statistically significantly higher than the equivalent size PDS™ Plus Suture knots at the 95% confidence interval as determined using a *t* test. The maximum average tensile for size 1 STRATAFIX™ SYMMETRIC PDS™ Plus Devices was 19.16 +/− 0.6 lbs as compared to PDS™ Plus suture at 14.28 +/− 0.7 lbs. Size 0 averaged 15.42 +/− 0.5 lbs as compared to PDS™ Plus suture at 10.14 +/− 0.7 lbs. Size 2/0 averaged 10.72 +/− 0.8 lbs. as compared to PDS™ Plus suture at 7.32 +/− 0.7 lbs and size 3/0 devices averaged 7.35 +/− 05 lbs as compared to PDS™ Plus suture at 5.84 +/− 0.4 lbs.

#### Fixation Tab Mass Comparison to Conventional Suture Knot Tower

Conventional suturing technique requires surgical knots to secure the proximal and distal ends of a continuous suture pattern and for each stitch of an interrupted suture pattern. Knots add foreign body mass to the implanted suture. The fixation tab of the STRATAFIX™ SYMMETRIC PDS™ Plus Device that secures the proximal end of the closure adds significantly less mass than traditional knots. The mass of a Size 1 STRATAFIX™ SYMMETRIC PDS™ Plus Device fixation tab was compared to a 5-throw surgical knot tower of Size 1 PDS™ Suture.

In order to obtain the knot towers, five throw knots were tied around a cylinder and the knot tower was cut away from the cylinder. The loop below the knot tower would be entirely within tissue in clinical use. The amount of suture comprising this loop is dependent on many variables such as needle geometry and tissue thickness. Therefore, the mass from the loop below the knot tower was not included in the measurements; only the knot tower itself and the short tag ends were weighed making it a conservative value. The fixation tabs were simply cut from the distal end of the STRATAFIX™ SYMMETRIC PDS™ Plus Device.

Ten samples per group were evaluated. The mass of the fixation tab is approximately one third of a 5-throw knot tower of the equivalent size suture. The STRATAFIX™ SYMMETRIC PDS™ Plus fixation tab averaged 0.00496 g as compared to PDS™ Plus 5 throw not tower of 0.01839 g. A *t* test was used to determine the difference is statistically significant at the 95% confidence interval.

#### Initiation Stitch Strength

A functional test was developed to mimic the motion and mechanics of the surgeon during initial placement of the STRATAFIX™ SYMMETRIC PDS™ Plus Device into tissue by measuring initiation stitch strength. This method includes seating the fixation tab into intact tissue as shown in Fig. 2a–c and then making a single point of closure within the incision. Axial load is applied directly to the device using the remaining length to determine initiation stitch strength. For control specimens prepared using PDS™ Plus suture, the initiation stitch is created by applying a 5-throw surgical knot to make a single point of closure within the incision. The remaining length of suture is used for axial loading.

Testing was conducted for Sizes 0 and 1 STRATAFIX™ SYMMETRIC PDS™ Plus Devices and PDS™ Plus suture in porcine midline abdominal (fascia) tissue. Cadaveric porcine tissue specimens were prepared by separating the subcutaneous fat and skin layer using a scalpel and retaining the muscle layer with midline fascia for applying the devices. Two small incisions (∼1.5 in. each) were made in the fascia midline to allow for four test locations per tissue specimen. The tissue beyond the incision ends was left intact in order to simulate an “apex” location for the initiation stitch.

The tissue specimen was secured in a proprietary fixture. Axial load was applied to the free end of the device or suture (loaded perpendicular to the fixed tissue plane) until a device or tissue failure was noted by the test operator. This set-up simulates a surgeon pulling ‘up’ on the free device or suture end after initiation into the tissue. The free end of the device or suture is fixed into the upper grip. Using a calibrated INSTRON™ Mechanical Testing Unit, specimens were loaded and pulled at a constant strain rate until rupture. Note: the fixture was weighted down to apply resistance to the linear load.

Ten samples per group were tested. The average maximum initiation strength of STRATAFIX™ SYMMETRIC PDS™ Plus Size 1 devices was 15.16 +/− 1.96 lbs as compared to 13.61 +/− 1.17 lbs for PDS™ Plus suture. For size 0, the average maximum initiation strength of STRATAFIX™ SYMMETRIC PDS™ Plus Size 0 devices was 13.89 +/− 0.4 lbs as compared to 8.34 +/− 1.62 lbs for PDS™ Plus suture.

The STRATAFIX™ SYMMETRIC PDS™ Plus Device achieved statistically higher maximum initiation stitch strength for both sizes 0 and 1 relative to the 5-throw PDS™ Plus knot. Regarding the failure modes for STRATAFIX™ SYMMETRIC PDS™ Plus Device, the size 1 devices have a stronger, more robust core than the size 0 devices and thus experienced more fixation tab failures. For PDS™ Plus, failure occurred primarily at the surgical knot, regardless of size. Surgical knots introduce stress concentrations into the strand, which greatly reduce their inherent tensile strength. Tissue tearing may occur in this ex vivo model and was noted to occur for PDS™ in the same measured range as device failure.

The results of measuring initiation stitch strength are valuable in verifying the integrity of the STRATAFIX™ SYMMETRIC PDS™ Plus device. The fixation tab’s placement into intact tissue positively impacts the performance of the device when beginning an incision closure by counteracting the direct and upward loading on the device to ensure secure initiation

**Table 1:** Maximum Wound Holding Strength of STRATAFIX™ SYMMETRIC PDS™ Plus Devices and PDS™ Plus Loop, PDS™ Plus Suture and Coated VICRYL™ Plus Suture *n* = 10 samples per group

Size	Avg. Maximum Wound Holding Strength (lbs) +/− Std Dev. STRATAFIX™ SYMMETRIC PDS™ Plus Devices (95% CI)	Avg. Maximum Wound Holding Strength (lbs) +/− Std Dev. of PDS™ Plus Loop or PDS™ Plus Suture (95% CI)	Avg. Maximum Wound Holding Strength (lbs) +/− Std Dev. of Coated VICRYL™ Plus Suture	*p* value
2–0 Subcutaneous tissue	90.06 +/− 16.38 (73.68, 106.44)	86.32 +/− 18.62 (67.70, 104.94)	Not tested	0.64
0 Abdominal wall tissue	129.50 +/− 17.88 (111.62, 147.38)	109.79 +/− 31.84 (77.95, 141.63)	93.42+/−22.02 (63.68, 128.34)	0.009

.

#### Wound Holding Strength Table 1

Wound closure integrity is a critical performance parameter during the wound healing process. The ability of any wound closure device to maintain wound approximation during healing is a function of device strength and placement technique, tissue integrity, and peripheral loading of the incision site. The factors inherent to the device are controlled primarily during application at the time of surgery. Tissue health and loading are unique to each patient and can be challenging to predict and mitigate post-surgery.

In order to assess wound holding performance under simulated physiologic load, a porcine survival model was developed. The robust porcine abdominal tissue is a well-established model that mimics high tension surgical closures in the human abdominal wall and fascia tissue layers. The model allows for device application along the linea alba, a secure fixture of the ex vivo test specimen, and stable axial tensile strain until rupture of the incision site. Benchtop assessments were validated against data collected using a human cadaver specimen in order to ensure holding strength values are on a comparable scale.

Fresh cadaveric porcine tissue was procured from a local vendor. Specimens were full thickness ventral slabs large enough to dissect and re-approximate; typically10 × 10 in. in size. Slab thickness varied between animals yet linea alba thickness was consistently 0.4–0.6 cm. A scalpel was used to separate the abdominal wall tissue from the subcutaneous layer (and skin layer).

The abdominal wall tissue specimen was incised along the linea alba with a scalpel, creating a 10 cm length incision with intact tissue beyond the apexes. A stainless steel template was used to mark the area intended for closure. Lateral spacing between bites was 1 cm and medial to the incision was 1 cm from incision edge. This spacing represents the natural curvature and length of a surgical needle used for abdominal closures. Size 0 devices were used in this tissue layer.

The subcutaneous tissue specimen was incised to create a similar 10 cm incision with intact tissue beyond the apexes. The same marking template was used on these specimens. Size 2–0 devices were used in this tissue layer. The STRATAFIX™ SYMMETRIC PDS™ Plus Devices were applied in the prepared porcine tissue following the steps outlined in the Intra-Operative Usage description. The fixation tab was seated into intact tissue beyond the proximal incision apex and secured with a single pass. The device was applied in a continuous pattern to close the (marked) 10 cm incision. At the distal apex, the device was passed back over the incision closure two times, creating a “double reverse” locking stitch as shown in Fig. 2e. The device was trimmed at the level of the tissue plane to complete the closure.

Size 0 PDS™ Plus Looped Suture closures were made in the abdominal wall tissue using a continuous pattern following the same 1 cm spacing scheme and secured on the proximal end by the suture loop and by a 5-throw knots on the distal end; two surgeon’s knots and an extra throw. The PDS™ Plus Suture Size 2–0 devices used in the subcutaneous tissue were not looped and were instead secured on either incision end using 5-throw knots as described.

Size 0 Coated VICRYL™ Plus Antibacterial (polyglactin 910) Suture closures were made in the abdominal wall tissue using a continuous pattern following the same 1 cm spacing scheme and secured on the proximal and distal ends using 5-throw knots as described. These incision closures are intended to mimic high-tension abdominal wall closures over time. These closures are appropriate for suture sizes 2–0 and larger and are compatible with either continuous or interrupted suture closure techniques.

Using a fabricated test fixture, specimens were loaded axially on an INSTRON™ Mechanical Testing Unit) at a constant strain rate until tissue or device failure was observed by the operator. Clamp distance was set at the same distance medial to the incision closure for each specimen. The maximum load at failure and the failure description were recorded for each specimen. Ten samples per group were tested.

As with the maximum initiation strength test, the failure modes were also assessed in the wound holding strength testing. In subcutaneous tissue, six samples of size 2/0 STRATAFIX™ SYMMETRIC PDS™ Plus failured by suture break and the remainder by tissue gapping. PDS™ Plus sutures had nine knot failures and one tissue failure. In abdominal wall tissue, size 0 STRATAFIX™ SYMMETRIC PDS™ Plus Devices all had tissue failure whereas the PDS™ Plus sutures all failed for suture break. Size 0 VICRYL™ Plus Suture had six failures for tissue gapping, three for tissue failure and one for suture break.

A one-way ANOVA was used to determine if the difference is statistically significant at the 95% confidence interval for the Size 0 devices in abdominal tissue. The STRATAFIX™ SYMMETRIC PDS™ Plus Device displayed statistically superior maximum tissue holding forces to both Coated VICRYL**™** Plus Suture and PDS™ Plus Looped Suture. The STRATAFIX™ SYMMETRIC PDS™ Plus Device also demonstrated a lower (14%) coefficient of variation (COV) than the comparators (24% VICRYL™ and 31% PDS™ Loop). The relatively low COV of the STRATAFIX™ SYMMETRIC PDS™ Plus Device may be due to there being only one failure mode. This indicates consistency between samples.

A *t* test was used to determine if the difference is statistically significant at the 95% confidence interval for the Size 2–0 devices in subcutaneous tissue. The STRATAFIX™ SYMMETRIC PDS™ Plus Device displayed statistically equivalent maximum tissue holding forces compared to PDS™ Plus Suture size 2–0. These results are valuable to validate the performance of STRATAFIX™ SYMMETRIC PDS™ Plus Devices when used in various tissue closures including those under significant loading. The wound holding capabilities are demonstrated to be as good as or better than conventional devices routinely used in soft tissue approximation.

## Conclusions

STRATAFIX™ SYMMETRIC PDS™ Plus Device is an absorbable, antibacterial, knotless tissue control device developed to facilitate soft tissue approximation by providing the performance characteristics and wound holding security of conventional PDS™ Plus Suture with added features that offer increased efficiency and control. The devices have increased tensile strength relative to the knot tensile strength of PDS™ Plus Sutures with comparable and sometimes superior wound holding strength in fascia. The advantages that these features provide over conventional suture are relevant and beneficial for obstetric/gynecologic surgeries. Further studies are needed to provide outcomes regarding safety, effectiveness, and adverse events.
